# Parental hesitancy toward children vaccination: a multi-country psychometric and predictive study

**DOI:** 10.1186/s12889-024-18806-1

**Published:** 2024-05-18

**Authors:** Hamid Sharif-Nia, Long She, Kelly-Ann Allen, João Marôco, Harpaljit Kaur, Gökmen Arslan, Ozkan Gorgulu, Jason W. Osborne, Pardis Rahmatpour, Fatemeh Khoshnavay Fomani

**Affiliations:** 1 Psychosomatic Research Center, Mazandaran University of Medical Sciences, Sari, Iran; 2 Department of Nursing, Amol School of Nursing and Midwifery, Mazandaran University of Medical Sciences, Sari, Iran; 3 Sunway Business School, Sunway University, Sunway City, Malaysia; 4 School of Educational Psychology and Counselling, Faculty of Education, Monash University, Clayton, Australia; 5 William James Centre for Research ISPA – Instituto Universitário, Lisboa, Portugal; 6 Business School, Taylor’s University Lakeside Campus, Subang Jaya, Malaysia; 7 Department of Psychological Counseling, Burdur Mehmet Akif Ersoy University, Burdur, Turkey; 8 Department of Biostatistics and Medical Informatics, Faculty of Medicine, Kırşehir Ahi Evran University, Kırşehir, Turkey; 9 Department of Statistics, Miami University, Oxford, OH USA; 10 School of Nursing, Alborz University of Medical Sciences, Karaj, Iran; 11 Nursing and Midwifery Care Research Center, School of Nursing and Midwifery, Tehran University of Medical Sciences, Tehran, Iran; 12 Centre for Wellbeing Science, Faculty of Education, University of Melbourne, Parkville, Australia

**Keywords:** Vaccine hesitancy, Psychometric, Nursing, Australia, China, Iran, Turkey

## Abstract

**Aim:**

Understanding vaccine hesitancy, as a critical concern for public health, cannot occur without the use of validated measures applicable and relevant to the samples they are assessing. The current study aimed to validate the Vaccine Hesitancy Scale (VHS) and to investigate the predictors of children’s vaccine hesitancy among parents from Australia, China, Iran, and Turkey. To ensure the high quality of the present observational study the STROBE checklist was utilized.

**Design:**

A cross-sectional study.

**Method:**

In total, 6,073 parent participants completed the web-based survey between 8 August 2021 and 1 October 2021. The content and construct validity of the Vaccine Hesitancy Scale was assessed. Cronbach’s alpha and McDonald’s omega were used to assess the scale’s internal consistency, composite reliability (C.R.) and maximal reliability (MaxR) were used to assess the construct reliability. Multiple linear regression was used to predict parental vaccine hesitancy from gender, social media activity, and perceived financial well-being.

**Results:**

The results found that the VHS had a two-factor structure (i.e., lack of confidence and risk) and a total of 9 items. The measure showed metric invariance across four very different countries/cultures, showed evidence of good reliability, and showed evidence of validity. As expected, analyses indicated that parental vaccine hesitancy was higher in people who identify as female, more affluent, and more active on social media.

**Conclusions:**

The present research marks one of the first studies to evaluate vaccine hesitancy in multiple countries that demonstrated VHS validity and reliability. Findings from this study have implications for future research examining vaccine hesitancy and vaccine-preventable diseases and community health nurses.

## Introduction

Emerging and re-emerging infectious diseases have threatened human life many times throughout history. Many researchers and experts agree that vaccinations are one of the most protective and preventative mechanisms for disease control and pandemic prevention [1]. For example, in case of COVID-19, vaccines were developed to boost immunity to curb the spread of the highly infectious disease [2] and save an estimated 14.4 million lives globally [3]. Despite the reported success of many vaccines in terms of disease spread, reduced symptoms, and adverse outcomes, as well as the historical success of vaccination more generally in preventing disease outbreaks, vaccine hesitancy remains an enduring and critical threat to health globally. Vaccine hesitancy has been identified as a central factor affecting vaccine uptake rates, impacting the potential emergence and re-emergence of vaccine-preventable diseases [4].

The SAGE Working Group on Vaccine Hesitancy defined vaccine hesitancy as a “delay in [the] acceptance or refusal of vaccination despite availability of vaccination services” and found that people’s reluctance to receive safe and available vaccines was a growing concern, long before the recent COVID-19 pandemic [5]. Previous research has linked vaccine hesitancy to various factors, such as concerns for safety and effectiveness, which may have emerged due to the unprecedented scale and speed at which the vaccines were developed [6]. Other factors fuelling vaccine hesitancy include a lack of information [7], conspiracy theories, and low trust in governments and institutions [8, 9].

### Parental vaccine hesitancy

Parental vaccine hesitancy is a crucial concern for public health due to its close links to vaccination delay, refusal, or denial in children, which ultimately increases their vulnerability to preventable diseases [10, 11]. It is estimated that approximately 25% of children aged between 19 and 35 months have not been vaccinated due to the vaccine hesitancy of their parents [12]. For parents specifically, hesitancy is associated with misinformation on the internet [13], concern for finances, skepticism towards vaccine safety and necessity, confidence in a vaccine, and perceptions of the vaccine’s risk [14]. Additionally, parental vaccine hesitancy may be influenced to a large extent by environmental conditions, such as epidemics. Accordingly, children’s vaccination was identified as a challenging health issue during the COVID-19 pandemic, with implications for the health and spread of the diseases to the broader population [15, 16].

Research has found that parental perceptions of risk and vaccine confidence generally contribute significantly to parental vaccine hesitancy. Parents have been reported to worry about potential side effects of the vaccines as well as their general effectiveness [12]. Meanwhile, low confidence in vaccination has been linked to reducing herd immunity and increasing infection among those who are immunocompromised or not vaccinated [17], especially in children.

### Theoretical perspectives

The Health Belief Model (HBM) proposed by Hochbaum, Rosenstock, & Kegels (1952) suggests that vaccine decision-making is based on individuals’ perceptions of diseases and vaccines. Therefore, the perceived severity and susceptibility of diseases and the perceived risks and benefits of the vaccines may predict parental intentions to vaccinate their children [18]. Parent decisions in protective behaviours can therefore be shaped by their appraisal of the threat. According to protection motivation theory (PMT), threat appraisal refers to one’s adaptive actions, which consist of threat severity, maladaptive rewards, and threat vulnerability [19]. Parental appraisals of a disease as a threat thus shape patterns of vaccine hesitancy.

Considering existing theories, models, and conceptualizations, various measures have been developed and evaluated for assessing vaccine hesitancy. These measures assess an individual’s confidence in vaccines (Vaccine Confidence Scale) [20, 21], parental attitudes toward childhood vaccines [22], and conspiracy beliefs related to vaccines [23]. Among the existing measures, the *Vaccine Hesitancy Scale* (VHS) was originally developed by Larson and colleagues from the SAGE Working Group on Vaccine Hesitancy [24], and psychometrically tested by Shapiro et al. (2016) among Canadian parents three years later. Their study revealed a two-factor structure (lack of confidence and risk) of the 9-item VHS among Canadian parents in French and English. In the study, one item was removed, and two items were loaded in the “risk” dimension, with the other six loading in the lack of confidence dimension [25]. Another study among parents in Guatemala also revealed a two-factor solution where the 7-item VHS was a better fit than the 10-item scale [26]. Further research is needed to refine the scale and assess its validity in different countries and contexts. Understanding vaccine hesitancy cannot occur without the use of validated measures applicable and relevant to the samples they are assessing. The current study, therefore, aims to psychometrically evaluate the Vaccine Hesitancy Scale among parents in Australia, China, Iran, and Turkey.

## Methods

### Study design and participants

The data used in this study is part of a broader research project on identifying the leading factors of parental vaccine hesitancy. A methodological cross-sectional research design was employed to validate the VHS based on data from four countries (i.e., Australia, China, Iran, and Turkey). A survey was distributed to parents across four countries over eight weeks, between 8 August 2021 and 1 October 2021. The inclusion criteria for respondents’ eligibility were parents with at least one child aged 18 years or under. The minimum sample size for conducting the Confirmatory Factor Analysis (CFA) was based on the criteria of [1] bias of parameters estimates < 10%; [2] 95% confidence intervals coverage > 91%; and [3] statistical power > 80% [27]. A minimum sample size of 200 was found to be sufficient to achieve the required criteria. To ensure the sample would reflect a normative population variance, this study collected more than 300 responses from each country. Using a convenient sampling technique, this study collected a total of 6,073 samples across the four countries: Australia (2734), China (523), Iran (2447), and Turkey (369). The online questionnaire was created by Google Form and sent to participants via social platform such as WhatsApp, Telegram and national application.

### Measures

#### Sociodemographic characteristics

The data of parents’ sociodemographic characteristics such as age, gender, education level, living area, their perception regarding their economic status, and being active in social media were gathered using a sociodemographic form.

#### The vaccine hesitancy scale (VHS)

The VHS (ten-items) was originally developed by the SAGE Working Group on Vaccine Hesitancy, which is used to access parental vaccine hesitancy in their children. Although the original measure was not psychometrically evaluated by the original developers, it was later validated amongst a sample of Canadian parents [25]. The VHS has a validated two-factor structure: (1) lack of confidence (seven items; e.g., “Childhood vaccines are important for my child’s health”), and (2) risk (two items; e.g., “New vaccines carry more risks than old vaccines”). The scoring procedure for items in the VHS are rated on a 5-point Likert scale ranging from one (strongly disagree) to five (strongly agree). The current study consisted of four versions of the VHS: English (for Australia), Chinese (for China), Persian (for Iran), and Turkish (for Turkey). The English version was adopted from the Shapiro, Tatar [25] study. The Chinese, Persian, and Turkish versions were translated using the WHO protocol of forward-backward translation technique from the original English version. All versions were checked for cross-cultural equivalence.

### Translation procedure

The cross-cultural adaptation procedure [28] was used to translate the items (sociodemographic information and VHS) from English via the translation and back-translation procedure into Chinese, Persian, and Turkish. All translators were bilingual. Two translators independently translated the questionnaires into the country’s respective languages. The research team then assessed the translated versions selecting the most appropriate item translations. Following this step, two other bilingual translators, who were “blinded” to the original questionnaire version, conducted the back-translation procedure independently. The expert committee (consisting of research team members, two nurses, one physician in social medicine, and a methodologist) then checked the back-translated version to ensure the accuracy and equivalence to the original questionnaire version. The committee also assessed the cross-cultural equivalence and appropriateness of the questionnaire to the study population, as well as the semantic equivalence of the items. No items were changed during the procedure.

### Data analysis

#### Descriptive statistics

This study used R and RStudio to perform all statistical analyses. The *skimr* and *psych* package was applied to produce descriptive statistics, which included the minimum v (Min), maximum (Max), and average value (M) as well as skewness and kurtosis for each item. Additionally, this study also generated histograms for each item [29–31]. Multiple linear regression was used to predict parental vaccine hesitancy from gender, Self-perception as being an active person on social media, and perceived financial well-being.

#### Confirmatory factor analysis

This study conducted a confirmatory factor analysis (CFA) using the *lavaan* package to assess the psychometric properties of the VHS across four countries. The factorial structure and model fit was confirmed and assessed in this stage. Model fit was evaluated using several fit indices such as the comparative fit index (CFI) > 0.90, normed fit index (NFI) > 0.90, Tucker–Lewis’s index (TLI) > 0.90, Standardized Root Mean Square residual (SRMR) < 0.09, and root mean square error of approximation < 0.08 [32, 33].

#### Construct validity and reliability

To assess the VHS’s construct validity, both convergent and discriminant validity were assessed using the *SemTools* package. For convergent validity, the Average Variance Extracted (AVE) for each construct should be more than 0.5 [34]. Concerning discriminant validity, this study followed the Heterotrait-monotrait ratio of correlations (HTMT) approach, which denotes that all correlations between constructs in the HTMT matrix table should be less than 0.85 [35] and the correlations should have an AVE larger than the squared correlation between factors (Fornell & Larcker, 1981; Marôco, 2021). To assess the reliability of the VHS, the *SemTools* package was used to compute Cronbach’s alpha (α) and omega coefficients (ω), where α and ω values greater than 0.7 demonstrates an acceptable internal consistency and construct reliability [36–38].

#### Invariance assessment

To detect whether the factor structure of the VHS holds across the four countries, a set of nested models were defined and compared using the *lavaan* package with robust maximum likelihood estimation, namely, configural invariance model (no constraints), metric invariance model (constrained factor loadings between four countries), scalar invariance model (constrained loadings and intercepts), and structural invariance model (second order factor loadings constrained). Invariance was assessed using absolute ΔCFI and ΔRMSEA < 0.02. Invariance was assumed for ΔCFI < 0.01 and absolute ΔRMSEA < 0.02 [39, 40] between two nested models as described elsewhere [27].

### Ethical considerations

The Ethics Committee of Mazandaran University of Medical Sciences Research Ethics Committee approved the Ethical Considerations of this study (ethic code: IR.MAZUMS.REC.1401.064). In addition, all participants were informed of the purpose of the data collection, and questionnaires were distributed to the respondents only after they provided their consent to participate in the survey. Moreover, the respondents were ensured that their participation was on a voluntary basis and the confidentiality of all collected data was guaranteed.

## Results

### Participants’ demographic characteristics and mean (S.D.) of COVID-19 vaccine hesitancy

This study employed a cross-sectional, questionnaire-based research design. In total, 6,073 parents from Australia (2734), China (523), Iran (2447), and Turkey (369) completed the survey through an online questionnaire platform. According to the Table 1, the majority of respondent were female (84.15%) and between 20 and 40 years old (54.61%).

**Table 1:** Demographic characteristics of respondents & comparing mean (SD) of vaccine hesitancy among parents

Variable	Countr*y*	Australia (n = 2734)		China (n = 523)		Iran (n = 2447)		Turkey (n = 369)	
		**n(percent)**	**M (SD)**	**n(percent)**	**M (SD)**	**n(percent)**	**M (SD)**	**n(percent)**	**M (SD)**
Parents’ Gender	Female	2611 (95.5)	34.7 (6.1)	340 (65)	21.5 (4.2)	1990 (81.32)	36.1 (4.2)	170 (46.1)	34.0 (5.3)
	Male	116(4.2)	33.4 (6.5)	183 (35)	21.8 (5.2)	433 (17.69)	35.3 (5.2)	199(53.9)	34.1 (4.2)
	Other	6 (0.2)	37.7 (4.3)	0 (0)	0 (0)	24 (0.98)	34.4 (0)	0 (0)	0 (0)
		P = 0.064, F = 2.7		P = 0.547, F = 0.3		P = 0.001, F = 7.2		P = 0.718, F = 0.1	
Parents’ Age	< 20 years old	3 (0.1)	33.6 (6.6)	12(2.3)	19.6 (9.7)	24 (0.98)	35.8 (5.4)	0 (0)	-
	20–40 years old	1208 (44.2)	34.4 (6.3)	483 (92.3)	21.6 (4.4)	1508 (61.62)	35.9 (4.1)	118 (32)	34.0 (4.9)
	40–60 years old	1517 (55.5)	34.9 (6.0)	28 (5.4)	22.6 (4.6)	904 (36.95)	35.9 (4.2)	251 (68)	34.1 (4.7)
	60 and more	6 (0.2)	23.5 (7.5)	0 (0)	-	11 (0.45)	37.0 (2.7)	0 (0)	-
		P < 0.001, F = 8.1		P = 0.165, F = 1.8		P = 0.800, F = 0.3		P = 0.880, F = 0.0	
perceived activity level on social media	Active	2017 (73.7)	34.9 (6.0)	143 (27.3)	20.2(4.3)	1009 (41.2)	36.2 (3.9)	147 (39.8)	33.6 (5.1)
	passive	531 (19.4)	33.8 (6.7)	297 (56.8)	22.1 (4.3)	1271 (51.9)	35.7 (4.3)	175 (47.4)	34.2 (4.6)
	Not-sure	186(6.8)	33.9(6.1)	83(15.9)	22.4(5.2)	167(6.8)	35.6(4.4)	47(12.7)	35.1(3.7)
		P < 0.001, F = 8.4		P < 0.001, F = 10.0		P = 0.01, F = 4.3		P = 0.14, F = 1.9	
Perceived Financial well-being	Low	171 (6.3)	32.9(6.9)	30 (5.7)	20.5 (7.7)	177 (7.2)	35.7 (4.4)	8 (2.2)	35.2 (2.1)
	Moderate	1960 (71.7)	34.6(6.1)	288 (55.1)	21.7 (4.3)	1171(47.9)	35.7 (4.3)	289 (78.3)	33.8 (4.8)
	Good	603 (22.1)	35.2 (6.1)	205 (39.2)	21.7 (4.3)	1099 (44.9)	36.2 (3.9)	72 (19.5)	35.1 (4.9)
		P < 0.001, F = 8.1		P = 370, F = 0.9		P = 0.025, F = 3.7		P = 0.089, F = 2.4	

### Item distribution properties

Table 2 shows the descriptive summary of the nine items’ minimum value (Min), maximum value (Max), average value (M), skewness, kurtosis, and histograms. The Item number 10 was dropped out due to the cross loading.

**Table 2:** Distribution properties and factor loadings of VHS’s items (*N* = 6073)

Item	M	SD	skewness	kurtosis	Histogram	Factor loadings			
						China	Iran	Australia	Turkey
**VHS1**: Childhood vaccines are important for my child’s health	4.17	1.165	-1.436	1.080	▁▁▁▃▇	0.72	0.67	0.93	0.91
**VHS2**: Childhood vaccines are effective	3.957	1.122	-1.067	0.420	▁▂▃▇▇	0.75	0.70	0.92	0.95
**VHS3**: Having my child vaccinated is important for the health of others in my community	4.216	1.090	-1.471	1.413	▁▁▁▃▇	0.66	0.73	0.92	0.77
**VHS4**: All childhood vaccines offered by the government program in my community are beneficial	4.003	1.199	-1.085	0.155	▁▂▂▅▇	0.68	0.72	0.89	0.79
**VHS5**: New vaccines carry more risks than older vaccines	3.811	1.215	-0.764	-0.398	▁▂▅▆▇	0.45	0.37	0.61	0.65
**VHS6**: The information I receive about vaccines from the vaccine program is reliable and trustworthy	3.448	1.113	-0.516	-0.321	▂▂▆▇▃	0.67	0.58	0.80	0.34
**VHS7**: Getting vaccines is a good way to protect my child/children from disease	3.877	1.109	-1.027	0.328	▁▂▂▇▆	0.70	0.80	0.93	0.58
**VHS8**: Generally I do what my doctor or health care provider recommends about vaccines for my child/children	2.765	1.082	0.123	-0.494	▃▅▇▃▂	0.65	0.67	0.82	0.55
**VHS9**: I am concerned about serious adverse effects of vaccines	2.768	1.198	0.154	-1.044	▅▇▅▆▂	0.69	0.97	0.83	0.68

### Confirmatory factor analysis

**Fig. 1:**
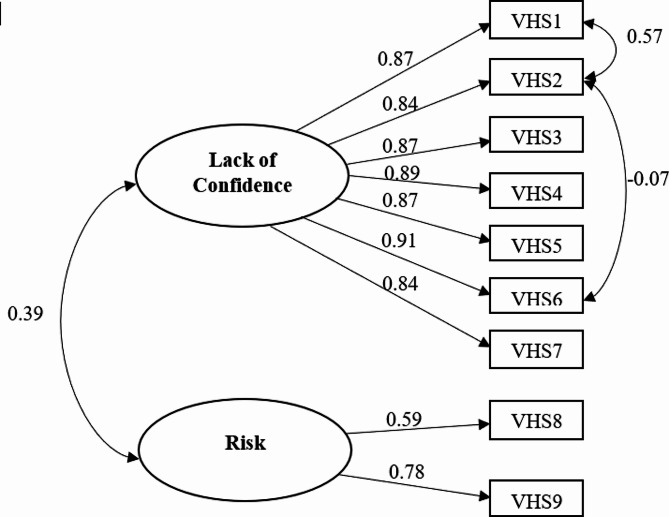
The results of the Confirmatory Factor Analysis (CFA)

A CFA was used to confirm whether the factorial structure of the VHS used in the current study was consistent with results from the original validation study. The results of the CFA demonstrated a good model fit of the two-factor measurement model as evidenced by the model fit indices: CFI (0.972), NFI (0.971), TLI (0.958), SRMR (0.037), and RMSEA (90% C.I.) [0.074 (0.067, 0.074)]. The results also showed that all factor loadings for all items were greater than 0.5 and statistically significant. Figure 1 depicts the factor structure of the VHS in this study.

### Construct validity assessment

The results showed that the AVE for the sub-factor “lack of confidence” was greater than 0.5 (0.735), and the AVE for the sub-factor “risk” was slightly less than 0.5 (0.494). Previous literature indicated that AVE is a conservative and strict measure of convergent validity, and convergent validity can be assessed on the basis of Composite Reliability (C.R.) alone. Therefore, based on the results of C.R., the VHS in this study established convergent validity across all countries. The results of the HTMT correlation matrix showed that discriminant validity was also achieved, as the HTMT between “lack of confidence” and “risk” was 0.395, which is less than the suggested cut-off value of 0.85. The squared correlation between the two factors was 0.153. As this factor is less than the AVE for both “lack of confidence” (0.735) and “risk” (0.494), further evidence of discriminant validity was supported.

### Construct reliability assessment

The results showed that the measurement model displayed good internal consistency and reliability, as evidenced by α (Lack of confidence: 0.952; Risk: 0.628) and ω (Lack of confidence: 0.946; Risk: 0.651).

#### Country invariance assessment

Prior to the Country Invariance Assessment, the vaccine hesitancy among parents score was compared across four countries. The results showed that the vaccine hesitancy among parents score was respectively in Iran (35.96, SD = 4.19), Australia (34.68, SD = 6.21), Turkey (34.09, SD = 4.78) and China (21.65, SD = 4.61) (*P* < 0.001). While China clearly has different average levels of parental vaccine hesitancy, this does not preclude similar psychometric properties (i.e., factor structure) to other countries.

Country invariance assessment was tested in line with standard procedures, with a set of nested increasingly constrained models (see Table 3).

**Table 3:** Analysis of invariance for countries

Model	AIC	BIC	χ^2^	df	Δχ^2^	Δdf	*p*	CFI	RMSEA	ΔCFI	ΔRMSEA
**Configural**	117665.1	118470.5	1169.84	96				0.972	0.086	0	0
**Metric**	118001.1	118665.5	1547.848	117	235.555	21	< 0.001	0.963	0.09	-0.009	0.004
**Scalar**	119107.5	119,631	2696.24	138	1044.521	21	< 0.001	0.933	0.11	-0.029	0.021
**Structural**	120548.1	121031.3	4148.859	144	1332.844	6	< 0.001	0.895	0.135	-0.038	0.025

First, configurational invariance tests whether the basic structure of the measure is invariant, imposing no equality restrictions on parameters. Second, metric (weak) invariance was tested by constraining factor loadings to be invariant across countries. The ignorable change from configural variance to metric invariance (ΔCFI and ΔRMSEA of -0.009 and 0.004 respectively) supports this level of invariance. Delta chi-squared was significant (Χ^2^_(21)_ = 235.55; *p* < 0.001), but chi squared is notoriously sensitive to ignorable changes when high *df* are present, and so is not considered a desirable metric.

Third, scalar invariance (“strong invariance”) constrained both factor loadings and item intercepts. Strong invariance is often considered beyond what is necessary for typical applications. These constraints produced a significant delta chi square (*Χ*^2^_(21)_ = 1044.251; *p* < 0.001) and a modest ΔCFI=-0.029; ΔRMSEA = 0.021. Finally, structural invariance, which constrained second-order factor loadings also produced a modest further degradation of model fit, but is also considered so extreme as to not be necessary. These results are sufficient to assert metric invariance.

*Predictive validity.* To further explore parental hesitancy, we examined whether VHS scores were related to gender, social media activity, and perceived financial well-being. All three variables, as predicted, were related to VHS. Because these variables were measured categorically, ANOVA was employed.

*Gender* was significantly related to VHS (*F*_*(1. 6070)*_ = 86.62, *p* < 0.001, *η*^2^ = 0.014), with those identifying as female or “other” having more vaccine hesitancy (M = 34.37, SD = 6.37; M = 34.04, SD = 6.53) than those identifying as male (M = 32.22, SD = 7.08).

*Social media activity* was significantly related to VHS (*F*_*(1. 5547)*_ = 69.54, *p* < 0.001, *η*^2^ = 0.012), with those indicating higher social media activity having more vaccine hesitancy (M = 34.89, SD = 5.86) than those indicating lower social media activity (M = 33.49, SD = 6.61).

*Financial well-being* was also modestly related to VHS (*F*_*(1. 6070)*_ = 42.52, *p* < 0.005, *η*^2^ = 0.002), with those identifying as most affluent having more vaccine hesitancy (M = 34.37, SD = 6.46) than those with moderate (M = 33.94, SD = 6.49) or low affluence (M = 33.32, SD = 7.12).

## Discussion

Vaccines reduce the diseases’ mortality and severity; therefore, vaccine hesitancy impacts global public health. The current study aimed to psychometrically evaluate the Vaccine Hesitancy Scale (VHS) among parents in Australia, China, Iran, and Turkey.

The current study found that a brief measure of parental vaccine hesitancy, when appropriately translated, is able to be used in broadly diverse sociocultural contexts. The Vaccine Hesitancy Scale showed strong and desirable psychometric properties, including predicted factor structure, strong reliability, metric invariance across country, validity, and expected relationships to self-reported outcomes such as affluence, gender, and social media engagement. These results align with the original validation study conducted in Canada [25] and another validating the scale in Guatemala [26].

These samples from four different countries and cultures were not ideal- there were far fewer fathers than mothers in three of the four samples (i.e., 4.2% of respondents in Australia, 35% in China, 17.69% in Iran, and 53.9% in Turkey were fathers). However, this could be considered a strength as in many cultures, mothers have more decision-making responsibility for the health and welfare of children than fathers [41], and it was mothers who were found to have higher vaccine hesitancy. This finding is aligned with the health belief model stating that gender plays a strong role in determining vaccine acceptance [18]. Existing qualitative research revealed the mothers’ mixed feelings on vaccination (e.g., confusion from conflicting information) [42]. Mothers in Australia expressed guilt about failing to be a good mother [43]. Studies have indicated that Chinese mothers exhibit a greater vaccine hesitancy for their children than fathers, due to their concerns regarding vaccine safety and effectiveness. It has been mentioned that fathers generally have a higher tendency for risk behaviours than mothers, so they may be more willing to vaccinate their children [44].

Among four countries, the vaccine hesitancy score was lower in China. It should be noted that this differences are not statistically significant. In China, parents are less hesitant to vaccinate their children compared to countries like Iran, Turkey, and Australia. This can be attributed to several key factors. Firstly, China has a communication strategy that focuses on transparency and providing authoritative information about vaccines, which has helped build public trust in the vaccination process. Additionally, China’s rapid development and distribution of COVID-19 vaccines have ensured a consistent supply of safe and effective vaccines, contributing to lower rates of vaccine hesitancy. Cultural and social factors also play a significant role, as China’s collectivist culture emphasizes community health and well-being, influencing parents to prioritize vaccinating their children. The Chinese government has implemented policies like providing free vaccines and launching public awareness campaigns to promote vaccination, reducing hesitancy rates. Moreover, China’s success in controlling infectious diseases through previous vaccination programs has created a positive attitude towards vaccines, influencing parents’ decisions. Overall, effective communication, safe vaccine availability, cultural influences, government initiatives, and past vaccination success have all contributed to lower levels of vaccine hesitancy among parents in China compared to other countries [14, 45].

Ancillary analyses observed age differences in vaccine hesitancy, but only in Australia, where parents between 40 and 60 years old were more vaccine hesitant than the other age groups (*p* < 0.001, F = 8.10), supporting past research [46] indicating that younger parents were less likely to be hesitant to vaccinate their children. The reason behind this phenomenon might be that younger parents have less experience with infectious diseases (such as smallpox and poliomyelitis) and, perhaps it makes them less hesitant to vaccinate their children against diseases.

Regarding the current study, the reason why older parents were more hesitant than younger parents might be that during the conducting of this study, they may have older children who should be vaccinated against COVID-19; the vaccine that its side effects, or even its effectiveness were not clear in this age group. When national health systems started to vaccinate children against COVID-19 older children were included in the program, and then it was extended to children five years old and older. It has been indicated that new vaccines generate more hesitancy [47]. further research needs to be conducted (e.g., qualitative research) to find more details.

These findings also noted that more affluent individuals, and those with more social media engagement tended to be more hesitant to their children’s vaccination, which aligns with prior studies [14, 48, 49]. Some prior studies have suggested that parents who perceived more financial comfort believed that their lifestyle could protect them from diseases, and therefore, they were more hesitant to vaccinate their children [49]. The role of social media on vaccine hesitancy has been identified by previous studies. In this regard, parents may be confused by misinformation and fake news in the media and on social networks [50]; consequently, they experience fear, stress, and a wide range of behavioural changes [51, 52]. Misinformation may make parents more cautious and force them to show their hesitancy with vaccines, especially new vaccines.

The current study indicated that lack of confidence in the vaccine and perceived vaccine risk contribute to parental vaccine hesitancy. According to the “3 Cs model” (confidence, complacency, and convenience) presented by the SAGE working group [53], lack of confidence in vaccine safety and effectiveness as well as low or mistrust of the systems that recommend or provide the vaccine can determine vaccine hesitancy. Furthermore, the model suggests that hesitancy may occur when parents do not value or perceive a need for vaccination (complacency) or when the vaccine is not accessible and available (convenience).

### Study limitations

This study has several limitations. First, the non-probabilistic samples enrolled in the current study could restrict the generalizability of the findings. Although the sample enrolled in the current study was large, convenience sampling may underrepresent certain population groups. Because these data were gathered using an online survey, findings may not generalize to those without access to electronic devices or the internet.

### Findings’ implications

This study supports broad use of this scale to evaluate parental vaccine hesitancy as part of an effort to understand and counteract resistance to adoption of vaccines in the general population. Applying this scale can provide valuable information for public health authorities to manage vaccine hesitancy among parents. The study indicated that women, those active on social media, and more affluent parents are more likely to resist having their children vaccinated, which can guide public health authorities in designing information campaigns to counteract these troubling trends. Healthcare providers can use this information to tailor their communication strategies to address the specific concerns of parents and increase vaccine uptake. Social media can play like a double-edged sword in parental vaccine hesitancy. Consequently, health policymakers are expected to do their best to provide authentic and accurate content that presents explicit information in the right way to the right audience.

## Conclusion

Parental vaccine hesitancy is prevalent globally and associated with several individual and contextual factors. It is estimated that vaccine hesitancy will become a major burden on public health worldwide. Without validated instruments in specific countries and contexts, it is not possible to conduct reliable and valid research to investigate the factors and determinants of parental vaccine hesitancy. The present study validated the Vaccine Hesitancy Scale (VHS) among parents in Australia, China, Iran, and Turkey during the COVID-19 outbreak. Acceptable psychometric evidence was found for the 9-item two-factor VHS using data from parents in four countries. Findings from this study have implications for future research examining vaccine hesitancy and vaccine-preventable diseases and community health nurses. Further studies are needed to test the scale’s validity and reliability across additional cultural contexts.

## Data Availability

The data used to support the finding of this study are available from the corresponding author upon reasonable request.
